# Oncogenic *KIT* mutations induce STAT3-dependent autophagy to support cell proliferation in acute myeloid leukemia

**DOI:** 10.1038/s41389-019-0148-9

**Published:** 2019-07-16

**Authors:** Clément Larrue, Quentin Heydt, Estelle Saland, Héléna Boutzen, Tony Kaoma, Jean-Emmanuel Sarry, Carine Joffre, Christian Récher

**Affiliations:** 1 Cancer Research Center of Toulouse (CRCT), UMR1037 INSERM, ERL5294 CNRS, Equipe Labellisée LIGUE, Toulouse, France; 2 University of Toulouse, Toulouse, France; 3 Proteome and Genome Research Unit, Department of Oncology, Luxembourg Institute of Health, Strassen, Luxembourg; 4 Service d’Hématologie, Centre Hospitalier Universitaire de Toulouse, Institut Universitaire du Cancer de Toulouse Oncopole, Toulouse, France

**Keywords:** Autophagy, Leukaemia

## Abstract

Autophagy is associated with both survival and cell death in myeloid malignancies. Therefore, deciphering its role in different genetically defined subtypes of acute myeloid leukemia (AML) is critical. Activating mutations of the KIT receptor tyrosine kinase are frequently detected in core-binding factor AML and are associated with a greater risk of relapse. Herein, we report that basal autophagy was significantly increased by the *KIT*^D816V^ mutation in AML cells and contributed to support their cell proliferation and survival. Invalidation of the key autophagy protein Atg12 strongly reduced tumor burden and improved survival of immunocompromised NSG mice engrafted with KIT^D816V^ TF-1 cells. Downstream of KIT^D816V^, STAT3, but not AKT or ERK pathways, was identified as a major regulator of autophagy. Accordingly, STAT3 pharmacological inhibition or downregulation inhibited autophagy and reduced tumor growth both in vitro and in vivo. Taken together, our results support the notion that targeting autophagy or STAT3 opens up an exploratory pathway for finding new therapeutic opportunities for patients with CBF-AML or others malignancies with *KIT*^D816V^ mutations.

## Introduction

Autophagy is an adaptive and protective cellular program activated during nutrient deprivation, growth factor withdrawal, or metabolic stress to maintain cellular homeostasis and recycle damaged organelles^1^. This dynamic process involves the rearrangement of subcellular membranes in order to sequester organelles or long-lived proteins and to then release these contents into the lysosomal machinery for degradation and recycling.

Defects in autophagy are associated with various diseases including cancer^2^. The role of autophagy in cancer is complex and depends on the tumor subtype, the stage of tumor progression, cellular context, and/or the drugs that induce this process^3,4^. Indeed, autophagy may suppress cancer initiation^5,6^ while enabling cell survival and growth of aggressive cancers, particularly during chemotherapeutic stress^7^. Oncogenes, such as mutant *Kras*^G12D^ or *Braf*^V600E^, have a high basal autophagy and need autophagy for growth^8,9^. In myeloid malignancies, BCR-ABL leukemic cells are also dependent on autophagy for cell survival and leukemogenesis^10,11^. Pharmacological inhibition of autophagy to potentiate BCR-ABL inhibitors is currently being investigated in chronic myeloid leukemia^12^.

It has recently been proposed that autophagy is required for leukemic development, as invalidation of *Atg7* or *Atg5* in a murine *Mll-Enl* leukemic model impaired tumor growth^13^ and involved in resistance to chemotherapy^13,14^. Moreover, high levels of autophagy seem to be associated with poor prognosis in acute myeloid leukemia (AML) patients. Indeed, AML cells from patients with complex cytogenetic abnormalities vs. normal karyotype or classified in adverse-risk group compared with the intermediate- or favorable-risk AML groups display higher levels of autophagy^15^. Accordingly, the mutant receptor tyrosine FLT3-ITD, which is also associated with a poor outcome, supports an elevated autophagy flux^16^. In addition, autophagy represents a mechanism of chemoresistance in specific AML models. This is the case upon mTORC1/mTORC2 inhibitors^17^ or upon histone deacetylase inhibitors^18^. However, in other contexts, inducing autophagy could be detrimental to leukemic cells. For instance, autophagy induced by arsenic trioxide, all-*trans* retinoic acid, or bortezomib contributes to cell death through the degradation of oncoproteins such as PML-RARA or FLT3-ITD in AML cells^19,20^. Thus, elucidating the role of autophagy in genetically defined AML subtypes is critical.

*KIT* mutations are found in 20−40% of patients with core-binding factors (CBFs) AML. These include AML with a t(8;21)(q22;q22) or inv^16^(p13q22) chromosomal rearrangement, which generate *RUNX1T1-RUNX1* and *CBFb*/*MYHII* fusion genes^21^. These mutations are associated with higher incidences of relapse after intensive chemotherapy and are associated with a poor prognosis^22^. The most frequent *KIT* mutations are point mutations, insertions, or deletions in exons 8 and 17, which encode the activation loop in the kinase domain and an extracellular region of KIT, respectively. Mutated *KIT* induces constitutive activation of phosphoinositide 3-kinase (PI3K)/AKT, ERK, and STAT3 pathways, and cooperates with *RUNX1T1-RUNX1* to induce AML in mice^23^. As these cell signaling pathways interfere with autophagy, we herein report on our investigation into the role of autophagy in *KIT*-mutated AML cells.

## Results

### *KIT* mutations induce autophagy, which supports cell proliferation and survival in AML cells

**Fig. 1:**
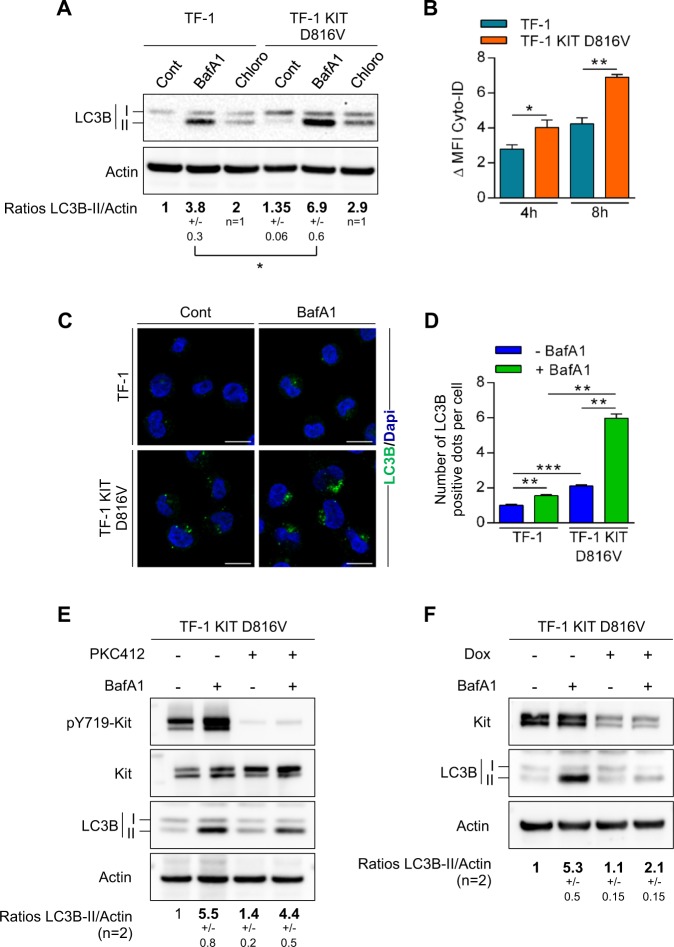
*KIT*^*D816V*^ mutation increases autophagic flux in AML cells **a**–**d** Oncogenic *KIT*^*D816V*^ drives autophagy. **a** TF-1 or TF-1 KIT^D816V^ cells were incubated for 4 h with PBS, Bafilomycin A1 (20 nM, *n* = 3 ± SEM), or chloroquine (20 µM, *n* = 2 ± SD), and were then analyzed by immunoblotting using the appropriate antibodies. Numbers represent the LC3B-II/actin ratios obtained by densitometric analysis. **b** Cells were incubated for 4 or 8 h with vehicle or 20 µM of chloroquine, and then incubated with Cyto-ID before flow cytometry (*n* = 3 ± SEM). ΔMFI = MFI(chloroquine) − MFI(vehicle). **c** Cells were treated with vehicle or BafA1 for 2 h before LC3 staining, fluorescent labeling, and immunofluorescence analysis. **d** Quantification of LC3-positive autophagosomes (*n* = 3 ± SEM). **e**, **f** Pharmacological inhibition or genetic invalidation of a decrease in KIT autophagic flux. **e** TF-1 KIT^D816V^ cells were treated overnight with PKC412 at 1 µM; 20 nM of BafA1 was added at 2 h before cell lysis and western blotting. Numbers represent the LC3B-II/actin ratios obtained by densitometric analysis (*n* = 2 ± SD). **f** KIT^D816V^ cells were incubated for 3 days with doxycycline to induce expression of shRNA against KIT. Cells were treated for 2 h with BafA1 (20 nM) and then analyzed by western blotting. Numbers represent the LC3B-II/actin ratios obtained by densitometric analysis (*n* = 2 ± SD)

We first compared basal autophagy in a TF-1 leukemic cell line that constitutively expressed wild-type *KIT* and in TF-1 engineered to express a *KIT-D816V* mutant (TF-1 KIT^D816V^). During autophagy, the microtubule-associated protein-1 light chain 3 (LC3-I) is converted to membrane-bound LC3-II and specifically associates with autophagosomes^24^. In order to address autophagic flux in cells harboring a *KIT*^D816V^ mutation, cells were incubated with lysosomal protease inhibitors, chloroquine, or Bafilomycin A, and then stained with cyto-ID or processed using western blotting analysis and LC3-I/II expression. Upon treatment of these autophagy inhibitors, both methods showed increased cyto-ID staining or LC3-II expression, which was more pronounced in TF-1 KIT^D816V^ compared with TF-1 cells and was consistent with active autophagic flux rather than a defect in autophagosome–lysosome fusion (Fig. 1a, b). Moreover, immunofluorescence microscopy showed accumulation of LC3-positive structures that was significantly enriched in TF-1 KIT^D816V^ compared with TF-1 cells (Fig. 1c, d). KIT^D816V^ was then pharmacologically inhibited by PKC412 (midostaurin) or was genetically invalidated by inducible short hairpin RNA (shRNA). Both methods demonstrated that targeting KIT^D816V^ strongly decreased autophagic flux (Fig. 1e, f).

**Fig. 2:**
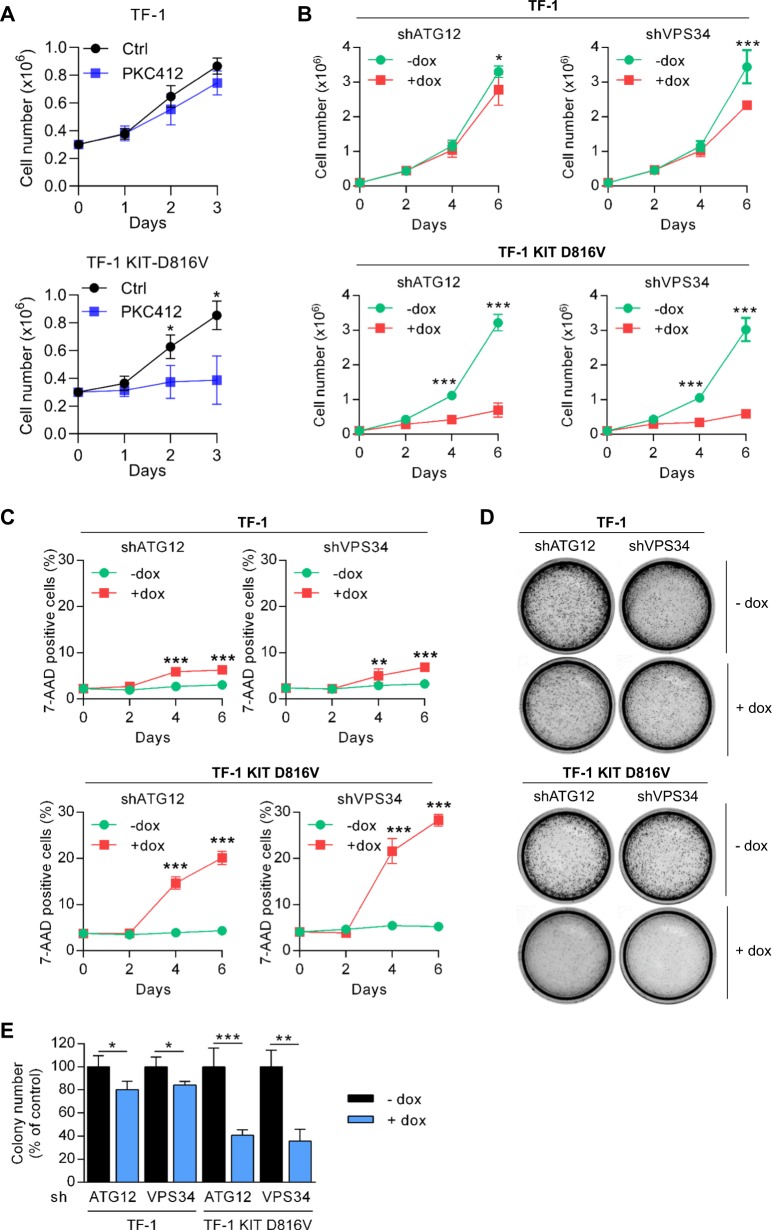
KIT-induced autophagy sustains cell proliferation and cell survival. **a** Impact of pharmacological inhibition of KIT on cell proliferation. TF-1 and TF-1 KIT^D816V^ cells were treated with PKC412 at 1 µM for 3 days and cell proliferation was evaluated by Trypan Blue exclusion counting (*n* = 3 ± SEM). **b**–**e** Consequences of invalidation of ATG12 and VPS34, two key autophagic proteins. TF-1 and TF-1 KIT^D816V^ cells were transduced with inducible shRNA against ATG12 or VPS34. Target proteins were invalidated after treatment with doxycycline (1 µg/mL). Effects of shRNA on cell proliferation (**b**), measured by Trypan blue exclusion (*n* = 3 ± SEM) and cell death (**c**) assessed after 7-AAD staining and flow cytometry (*n* = 3 ± SEM). **d**, **e** Colony-forming cell (CFC) assays. **d** shRNA-expressing cells were grown in methylcellulose medium in the presence of vehicle or doxycycline (5 µg/mL). **e** Quantification of colonies (*n* = 3 ± SEM)

To further investigate the role of autophagy in this model, we transduced TF-1 and TF-1 KIT^D816V^ with lentiviral vectors encoding inducible shRNA against Atg12 or Vps34, two key autophagic proteins. As expected, downregulation of Atg12 and Vps34 by shRNAs was associated with inhibition of KIT^D816V^-induced autophagy as shown by decreased LC3-II accumulation or LC3-positive structures (Supplementary Fig. S1A–C). Moreover, we showed that the pharmacological inhibition of KIT by PKC412 significantly reduced the proliferation of KIT^D816V^ expressing TF-1 cells without impacting the TF-1 cells number (Fig. 2a). This result indicates that the mutated cell line is dependent on KIT activation to proliferate. We then investigated the functional consequence of KIT^D816V^-induced autophagy on KIT^D816V^-dependent proliferation and survival by comparing the impact of Atg12 and Vps34 depletion in TF-1 and TF-1 KIT^D816V^ cell lines. Interestingly, although the invalidation of Atg12 and Vps34 had a small impact on TF-1 cell proliferation, significant inhibition of cell proliferation and induction of apoptosis were observed in TF-1 KIT^D816V^ cells (Fig. 2b, c). Moreover, the inhibition of the KIT^D816V^-dependent autophagy resulted in a strong reduction of the clonogenic properties of the mutated cell line in semi-solid culture (Fig. 2d, e). We also used AML cell lines that harbored the *KIT*^N822K^ mutation (SKNO-1) or no mutation (U937). The SKNO-1 cell line constitutively expressed a high basal LC3-II level and downregulation of Atg12 and Vps34 by shRNA inhibited cell proliferation and the clonogenic properties (Supplementary Fig. S2A–F). In contrast, in the U937 AML cell line, Atg12 or Vps34 invalidation had no impact on cell proliferation (Supplementary Fig. S2A–D). Altogether, these results demonstrate that *KIT* mutations induce autophagy that contributes to cell survival and proliferation in AML cells.

### Invalidation of Atg12 reduces tumor burden in vivo and prolongs mouse survival

**Fig. 3:**
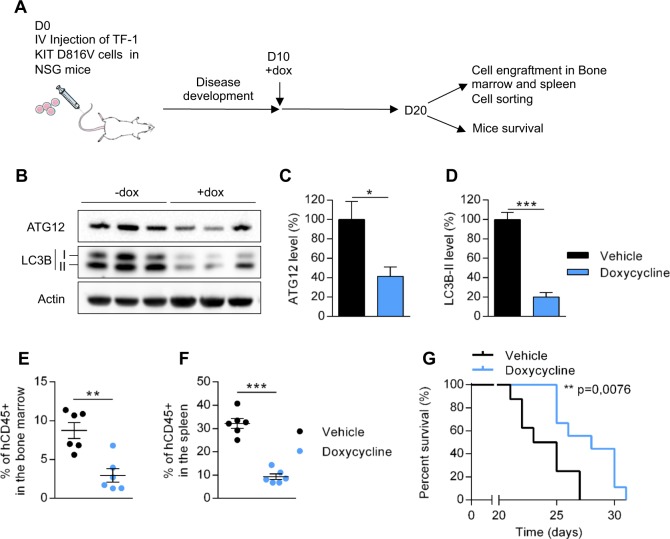
Targeting autophagy decreased leukemic development of human KIT^D816V^ cells when xenografted into NSG mice. A total of 2 × 10^6^ of TF-1 KIT^D816V^ cells expressing inducible shRNA against ATG12 were injected into the vein tails of NSG mice and doxycycline was added on day 10. On day 20, six mice per group were killed: the human cells were studied by flow cytometry and sorted. Overall mouse survival was also monitored. **a** Experimental procedure. **b** After killing the mice, human leukemic cells were isolated with hCD45 magnetic beads and analyzed by western blotting. **c**, **d** Quantification of protein level of ATG12 and LC3-II. Numbers represent the LC3B-II/actin ratios obtained by densitometric analysis (*n* = 3 ± SEM). **e**, **f** Percentage of human hCD45+ cells in the bone marrow (**e**) and spleen (**f**) of mice evaluated by flow cytometry (*n* = 6 ± SEM). **g** Overall survival of mice (vehicle *n* = 8, doxycycline *n* = 9)

To assess the role of autophagy in vivo, we used immunocompromised NSG mice engrafted with TF-1 KIT^D816V^/shAtg12 cells by intravenous injection. After establishment of the disease, mice were treated with vehicle or doxycycline to induce Atg12 downregulation and to inhibit autophagy (Fig. 3a). Ten days after doxycycline treatment, the levels of Atg12 and LC3-I/II were strongly downregulated (Fig. 3b–d). The selective knockout of Atg12 was associated with a strong decrease of tumor cell burden when compared with vehicle treatment, as indicated by the reduced percentage of human leukemic cells (hCD45 + ) in murine bone marrow and the spleen (Fig. 3e, f). Furthermore, downregulation of Atg12 significantly prolonged mouse survival (*p* = 0.0076) (Fig. 3g).

To confirm these results in another model, we used murine Ba/F3 cells that stably expressed hKIT^WT^ or hKIT^D816V^. A higher autophagic flux was observed in cells harboring hKIT^D816V^ mutation compared with hKIT^WT^ cells (Supplementary Fig. S3A). Using inducible shRNA against Atg12, which reduced LC3B-II level (Supplementary Fig. S3B), we showed that inhibition of autophagy significantly decreased cell proliferation in vitro and leukemic progression in vivo of Ba/F3-KIT^D816V^ cells (Supplementary Fig. S3C–D). Thus, these results demonstrate that KIT^D816V^-induced autophagy is necessary to drive leukemic progression in vivo, and that autophagy inhibition could represent a valuable therapeutic target in AML with *KIT* mutations.

### *KIT* mutant induces autophagy through STAT3 activation in AML cells

**Fig. 4:**
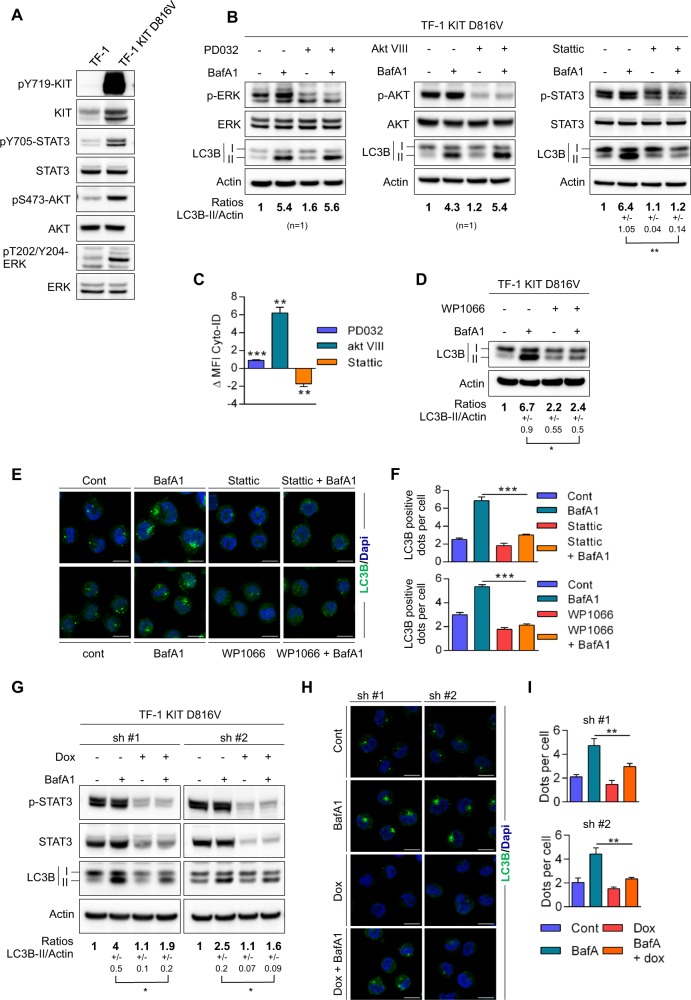
STAT3 drives autophagy in KIT^D816V^ cells. **a** Comparison of cell signaling in TF-1 and TF-1 KIT^D816V^ cells. **b** Identification of the signaling pathway involved in KIT^D816V^-induced autophagy. TF-1 KIT^D816V^ cells were treated for 2 h with PBS and BafA1 at 20 nM alone or in association with the indicated inhibitors. PD0325901 was used at 100 nM (*n* = 1), Akt inhibitor-VIII at 1 µM (*n* = 1), and Stattic at 10 µM (*n* = 3 ± SEM). The cells were then lysed and analyzed by immunoblotting. Numbers represent the LC3B-II/actin ratios obtained by densitometric analysis. **c** Assessment of autophagic flux by flow cytometry after Cyto-ID labeling. Cells were incubated with 10 µM of chloroquine alone or in combination with PD03259, Akt inhibitor-VIII, or Stattic, as previously described. ΔMFI = MFI(chloroquine + inhibitor) – MFI(chloroquine) (*n* = 3 ± SEM). **d** STAT3 inhibitor WP1066 was added at 15 µM alone or in combination with 20 nM of BafA1 at 2 h before western blotting. Numbers represent the LC3B-II/actin ratios obtained by densitometric analysis (*n* = 3 ± SEM). **e** Cells were treated for 2 h with vehicle, BafA1, Stattic, or WP1066 alone or in combination with BafA1 before LC3 staining, fluorescent labeling, and immunofluorescence analysis. **f** Quantification of LC3-positive autophagosomes (*n* = 3 ± SEM). **g**–**i** Genetic invalidation of STAT3. TF-1 KIT^D816V^ cells were transduced with two different inducible shRNAs against STAT3. Doxycycline (1 µg/mL) was added 3 days before the experiment. Autophagic flux was assessed by western blotting **g** with numbers representing the LC3B-II/actin ratios obtained by densitometric analysis (*n* = 3 ± SEM) and by immunofluorescence (**h**) on cells treated ±dox and ±20 nM BafA1. **i** Quantification of LC3-positive autophagosomes (*n* = 3 ± SEM)

The oncogenic properties of KIT^D816V^ are mediated by constitutive activation of STAT3/5, mitogen-activated protein kinase (MAPK), and PI3K/AKT pathways. As these signaling pathways modulate autophagy, we sought to determine which downstream target of KIT^D816V^ drives autophagy in this model. We first compared cell signaling in TF-1 cells and in TF-1 KIT^D816V^, and observed that, as expected, TF-1 KIT^D816V^ displayed constitutive phosphorylation of STAT3, ERK, and AKT compared with TF-1 cells (Fig. 4a). Interestingly, the wild-type KIT receptor, once activated by its ligand in both TF-1 and OCI-AML3 AML cells, induced, as observed in constitutively activated KIT^D816V^ mutant cells, (Supplementary Fig. S4A–E) autophagy and activation of STAT3, ERK, and AKT pathways (Supplementary Fig. S4F). We then assessed the impact of pharmacological inhibitors in these pathways on autophagic flux in TF-1 KIT^D816V^ cells and in cells expressing the wild-type KIT receptor upon its activation by the stem cell factor (SCF). Inhibition of ERK by PD0325901 had no impact on autophagic flux, whereas the AKT inhibitor increased it, likely through mammalian target of rapamycin (mTOR) inhibition (as expected; Fig. 4b, c and Supplementary Fig. S4G).

In contrast, Stattic, a small-molecule inhibitor of STAT3, significantly reduced the accumulation of LC3-II upon Bafilomycin A treatment in TF-1 KIT^D816V^ cells (Fig. 4b) but also in TF-1 or OCI-AML3 when stimulated with SCF (Supplementary Fig. S4GH), indicating a reduced autophagic flux upon STAT3 inhibition. Similar results were obtained using cyto-ID staining and immunofluorescence analysis (Fig. 4c–f and Supplementary Fig. S4G, IJ). These results were recapitulated with another STAT3 inhibitor, WP1066 (Fig. 4d–f and Supplementary Fig. S4H–J). We then transduced TF-1 KIT^D816V^ with lentiviral vectors encoding two inducible shRNAs against STAT3. In these cells incubated with Bafilomycin A, the selective knockdown of STAT3 decreased the conversion from LC3-I to LC3-II and the number of LC3-positive structures seen in immunofluorescence microscopy (Fig. 4g–i). Altogether, these data demonstrate that activated KIT, either constitutively or upon SCF stimulation, induces autophagy through STAT3 activation in AML cells.

To decipher the link between STAT3 activation and autophagy induction, we performed a transcriptomic analysis on TF-1 cells expressing either the wild type or the mutated form of KIT (Supplementary Fig. S5A). Consistently with the activation of STAT3 (Supplementary Fig. S5B), STAT3-regulated genes were found overexpressed in KIT^D816V^-expressing cells compared with wild-type cells (Supplementary Fig. S5A,C). Indeed, the expression of a previously identified STAT3 gene signature^25^ (DAUER_STAT3_TARGETS_UP) was significantly enriched as indicated by Gene Set Enrichment Analysis (GSEA) (Supplementary Fig. S5C). However, autophagy-related genes controlled by STAT3 were not differentially expressed between the two cell lines. Accordingly, the expression of two identified autophagy signatures (GO_REGULATION_OF_AUTOPHAGY; KEGG_REGULATION_OF_AUTOPHAGY) are not enriched in KIT^D816V^ cells and are rather enhanced in wild-type cells (Supplementary Fig. S5D). Together, these results are not in favor of a transcriptional regulation of autophagy by STAT3 in this model.

### In vivo targeting of STAT3 inhibits autophagy and reduces tumor cell burden in KIT^D816V^ AML cells

**Fig. 5:**
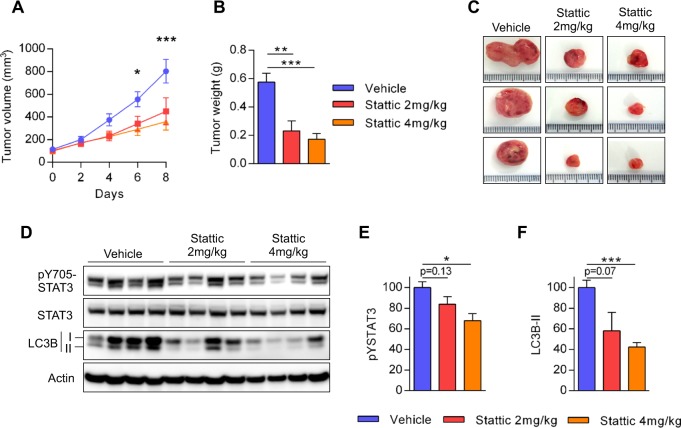
Pharmacological inhibition of STAT3 decreases tumor growth and autophagic level in vivo. TF-1 KIT^D816V^ (2 × 10^6^) cells were injected subcutaneously into NOD-SCID mice (*n* = 6 per group). When tumors reached 50−100 mm^3^ in size, the mice were treated daily with 100 µL of PBS or 100 µL of Stattic at 2 or 4 mg/kg/day. **a** Tumor growth was evaluated by measuring tumors with a caliper on the indicated days. **b**, **c** On the last day of the experiment, the tumors were dissected and weighed. **d**–**f** Targeting the effects of STAT3 on autophagy in vivo. **d** The tumors were ground, lysed, and then analyzed by western blotting using the indicated antibodies. **e**, **f** Quantification of western blotting (*n* = 4 ± SEM)

To inhibit STAT3 activity and to perform a preclinical proof-of-concept that STAT3 is an effective molecular target for AML cells expressing mutated KIT, we used the Stattic inhibitor directly injected into the tumor as previously described in vivo^26,27^. Therefore, we established a xenograft model using NOD/SCID mice that were subcutaneously injected with TF-1 KIT^D816V^ cells. After tumor establishment, the mice were treated with daily intra-tumoral injections of Stattic (2 or 4 mg/kg/day) or vehicle. Stattic treatment reduced STAT3 phosphorylation, strongly downregulated LC3-II expression, especially at the higher dosage, and significantly inhibited tumor growth. This indicates that pharmacological inhibition of STAT3 inhibits autophagy in vivo resulting in significant anti-leukemic activity (Fig. 5a–f).

## Discussion

Similar to other oncogenes, including *BRAF*^*V600E*^ or *KRAS*^*G12D*^ in solid tumors^8,9,28,29^ or *BCR-ABL* in chronic myelocytic leukemia^11^ and *FLT3-ITD* in AML^16^, our study shows that activating mutations of the tyrosine-kinase receptor KIT triggers autophagy and supports cell proliferation and survival in AML cells. Very recent insights into the AML cell metabolism have revealed that several metabolic pathways (e.g., glucose, glutamine, or fatty acid) regulated by autophagy, are crucial for AML cell growth and survival. Thus, RTK mutations, including *KIT* and *FLT3-ITD*, could activate autophagy to maintain a sufficient pool of energy substrates to sustain tumor growth and chemoresistance^30^. Indeed, it has been recently shown that glutamine metabolism is required for energy production through the tricarboxylic acid cycle (TCA) cycle and for redox homeostasis in AML cells^31,32^. Similarly, fatty acid uptake and high oxidative metabolism appear to be key players in the chemoresistance of AML^33–35^. Whether KIT-driven AML cells are preferentially dependent on specific metabolic substrates remains to be determined.

As far as hematological malignancies are concerned, autophagy has been mainly described as a mechanism of resistance to cytotoxic agents or tyrosine-kinase inhibitors, including cytarabine or imatinib^10,14^. It has been shown that following invalidation of the key autophagic genes, *ATG7* or *ATG5*, AML cells were sensitized to the anti-leukemic activity of cytarabine in vivo and prolonged mouse survival. Thus, it is reasonably conceivable that the introduction of new potent autophagic inhibitors into the armamentarium of anti-AML therapies could be of benefit in *KIT*-mutated AML^36–38^. It is also noteworthy that, in sharp contrast to imatinib or crizotinib that induce cytoprotective autophagy, midostaurin (PKC412), a pan-tyrosine-kinase inhibitor that potently targets KIT, decreased autophagy flux in *KIT*-mutated AML cells^10,39^. Midostaurin, which is active in advanced systemic mastocytosis with *KIT*^*D816V*^ mutations, was recently found to significantly increase overall survival in a phase-3 clinical trial on AML patients with *FLT3* mutations^40^. Our results suggest that CBF-AML with a *KIT* mutation should also benefit from midostaurin treatment through inhibition of cell signaling without activating cytoprotective autophagy.

STAT3 has been involved as a positive or negative regulator of autophagy, depending on the cellular context and its subcellular localization patterns^41^. On the one hand, it has been shown that cytoplasmic STAT3 repressed autophagy in osteosarcoma U2OS cells by inhibiting PKR activity^42^. In these cells, pharmacological inhibitors of STAT3, i.e., WP1066 or Stattic, strongly increased autophagy flux. On the other hand, some studies suggest that following phosphorylation on serine 727 by ERK or JNK kinases, STAT3 is translocated to the mitochondria where it supports cellular respiration and autophagy^43–47^.

In addition, STAT3 is a transcriptional enhancer of several autophagy-related genes in the nucleus and this activity contributes to a range of anti- or pro-autophagic functions of STAT3 in autophagy^41^. For example, it has been demonstrated that the expression of anti-apoptotic genes, *BCL2* and *MCL1*, are increased by STAT3^48,49^, whereas *BECN1* is downregulated^50^, resulting in anti-autophagic functions of STAT3. In contrast, STAT3 can enhance *HIF1A* or *BNIP3* expression, which is associated with an increase in autophagy^51,52^. More recently, interleukin (IL)-6-induced autophagy has been shown in hypoxic glioblastoma cells via the p-STAT3-MIR155-3p-CREBRF pathway^53^. *KIT*-activating mutations are associated with both Y705 and S727 phosphorylation of STAT3, leading to nuclear translocation and transactivation of target genes^54,55^. Thus, the pro-autophagic functions of STAT3 in our model could have been the result of this enhanced nuclear activity. However, our transcriptomic analysis (Fig. 5b–d) performed on cells expressing the mutated KIT is more in favor of a transcription-independent role of STAT3 on autophagy modulation. A function for cytoplasmic localized STAT3 is then more likely to be implicated and further studies are needed to fully assess the role of STAT3 in autophagy downstream of activated KIT.

Therapeutic approaches that exploit autophagy properties or target STAT3 represent a new field of investigation in hematological cancers. Classic pharmacological inhibitors of autophagy, including hydroxychloroquine, are being tested in combination with chemotherapy to treat AML, whereas new more specific compounds are currently being evaluated in early clinical trials^56^. Our study suggests that certain molecular subtypes of AML could be more susceptible to responding to inhibition of autophagy, thus indicating that results from clinical trials that are assessing autophagy inhibitors will need to take the genetic heterogeneity of AML into account.

## Materials and methods

### Cell lines

TF-1 and TF-1 KIT^D816V^ cell lines were a kind gift from Patrice Dubreuil laboratory (Marseille, France). TF-1 was grown in RPMI containing 10% fetal calf serum (FCS) supplemented with 2 ng/ml granulocyte macrophage colony-stimulating factor (GM-CSF) (Miltenyi Biotech, France). At 4 h before the experiments, cells were washed and maintained in GM-CSF-free medium. TF-1 KIT^D816V^ and OCI-AML3 cells were cultured in RPMI 1640 medium with Glutamax (Gibco, Life Technologies) supplemented with fetal bovine serum (Sigma) and 100 units/mL of penicillin and streptomycin (Invitrogen). Human myeloid-leukemia cell lines SKNO-1 and U937 were purchased from the Leibniz Institute DSMZ (the German Collection of Microorganisms and Cell Cultures, Leibniz, Germany) and cultured in RPMI containing 10% FCS and penicillin/streptomycin.

### Antibodies and reagents

An antiphospho-Kit (Y719), anti-Kit (D13A2), anti-LC3-B (for immunoblotting), antiphosphoStat-3 (Y705), antiphospho-Akt (S473), anti-Akt, antiphospho-p44/42 MAPK Erk1/2 (T202/Y204), and anti-p44/42 MAPK Erk1/2 were obtained from Cell Signaling Technology (Beverly, MA, USA); anti-STAT3 was obtained from Santa Cruz Biotechnology (CA, USA). For the immunofluorescence analysis, mouse monoclonal anti-Lc3-B (Nanotools, Germany) was used.

Secondary antibodies labeled with horseradish peroxidase were purchased from Promega. Stattic, PKC412, and WP1066 were purchased from Selleck Chemicals (Houston, TX, USA), Bafilomycin A1 was from Invivogen (Toulouse, France), and chloroquine and doxycycline hyclate were from Sigma (St. Louis, MO, USA). SCF was purchased from Miltenyi Biotech and used at 100 ng/ml concentration. The antibodies anti-hCD45 (YB5.B8, from BD Pharmingen), Annexin-V, 7-amino-actinomycin D (7-AAD) (Sigma), and a BD Via-Probe^TM^ (BD Pharmingen) were used in the flow cytometry studies.

### Western blotting analysis

Proteins were resolved using 4−12% *n*-polyacrylamide electrophoresis Bis-Tris gels (Life Technology, Carlsbad, CA, USA) and electro-transferred to nitrocellulose membranes. After blocking in phosphate-buffered saline (PBS)−0.1% Tween-20 and bovine serum albumin (BSA), the membranes were immunostained with appropriate antibodies and horseradish peroxidase-conjugated secondary antibodies. Immunoreactive bands were visualized by enhanced chemiluminescence (PI32209; Thermo Fisher Scientific) with a Syngene camera. Densitometric analyses of immunoblots were performed using GeneTools software.

### Cell-death assay

To assess cell death, 5 × 10^5^ cells were washed with PBS and then resuspended in 200 µL of PBS. 7-AAD (2 µL) was added at room temperature and in darkness. All samples were analyzed by a fluorescence-activated cell sorter (FACS: Calibur-Flow Cytometer, BD Pharmingen, San Diego, USA).

### Lentiviral infection of TF-1 cells

Lentiviral particles were generated by transient calcium-phosphate transfection in 293T cells. Briefly, 293T was placed in a 10 cm dish and transfected with 62.5 µL of CaCl_2_ (2 M), 500 µL of HeBS 2×, 418 µl of H_2_O, 3.5 µg of pVSV-G (env), 6.5 µg of p8.1 (tat, pol, rev, gag), and 10 µg of inducible TRIPZ human shRNA (GE Healthcare, CO, USA) against ATG12 (RHS4696-20075354), VPS34 (V3THS_372038), STAT3 (V2THS_262105 or V3THS_376016), or KIT (V2THS_76974). At 48 h after transfection of 293T, 2 mL of supernatant containing the lentiviral particles was incubated with ~2 × 10^6^ cells in six-well plates. Polybrene was added to make a final concentration of 8 µg/mL. Spinoculation was performed by centrifuging cells for 45 min at 800 × *g*.

On the day after transduction, the medium containing the virus was removed and changed for a virus-free medium. After an additional 24 h, the cells were selected using 1 µg/mL of puromycin. When puromycin-resistant cells appeared, the cells expressing a high level of shRNA (Red Fluorescent Protein-positive cells) were sorted by flow cytometry after 24 h of treatment with 1 µg/mL of doxycycline. All shRNA experiments were performed on bulk cells treated or not with 1 µg/mL of doxycycline for shRNA induction.

### Immunofluorescence microscopy

After the above treatments, the cells were placed onto poly-l-lysine-coated slides. Cells were then fixed in 4% formaldehyde for 8 min, and then for 5 min at 4 °C with 100% methanol. The cells were then permeabilized for 5 min with PBS containing 0.5% Triton X-100 and saturated for 30 min in PBS-5% BSA. The LC3B antibody (2 µg/mL), in PBS-5% BSA, was added 1 h at room temperature, followed by washing and 30 min of incubation in secondary antibody conjugated with Alexa-488, Alexa-555, or Alexa-647 (Invitrogen). Slides were mounted in Prolong Gold Antifade Mountant with 4′,6-diamidino-2-phenylindole (DAPI) (Invitrogen) and visualized on a Zeiss LSM710 or Zeiss LSM780 microscope using ZEN 2012 software. For quantification, fields were chosen arbitrarily based on DAPI staining and the number of LC3B dots per cell of at least 50 cells was determined using Image J software.

### Cyto-ID®

Autophagic flux was assessed using the Cyto-ID®-based procedure according to the manufacturer’s instructions (Enzo Life Sciences, Switzerland). The fluorescence of the Cyto-ID**®** dye incorporated into the different AML cells were analyzed by a FACS: Calibur-Flow Cytometer (BD Pharmingen, San Diego, USA; LSR Fortessa, BD Pharmigen).

### RNA microarray and bioinformatics analyses

Total RNA were extracted form TF-1 cells expressing either the wild-type KIT or the mutant KIT^D816V^ in independent triplicate using the Qiagen kit according to manufacturer’s instructions. RNA purity was monitored with NanoDrop 1ND-1000 spectrophotometer and RNA quality was assessed through Agilent 2100 Bionalyzer with RNA 6000 Nano assay kit. No RNA degradation or contamination were detected (RIN > 9). One hundred nanograms of total RNA were analyzed on Affymetrix GeneChip© Human Gene 2.0 ST Array using the Affymetrix GeneChip© WT Plus Reagent Kit according to the manufacturer’s instructions (Manual Target Preparation for GeneChip® Whole Transcript (WT) Expression Arrays P/N 703174 Rev. 2). Arrays were washed and scanned, and the raw files generated by the scanner was transferred into Partek© Genomics Suite for preprocessing (with default partek option). Quality control (boxplot, clustering and PCA) and differential expression analysis (with eBayes function, LIMMA package) were performed in R/CRAN environment. Mapping between transcript clusters and genes were done using annotation provided by Affymetrix (HuGene-2_0-st-v1.na34.hg19.transcript.csv) and the R/Bioconductor package hugene2.0sttranscriptcluster.db. *p*-values generated by the eBayes function were adjusted to control false discovery using the Benjamini and Hochberg’s procedure [RMA] Irizarry et al., Biostatistics, 2003; [Oligo package] Carvalho and Irizarry, Bioinformatics, 2010; [LIMMA reference] Ritchie et al., Nucleic Acids Research, 2015; hugene20sttranscriptcluster.db:MacDonald JW 2017, Affymetrix hugene20 annotation data (chip hugene20sttranscriptcluster); [FDR]: Benjamini et al., Journal of the Royal Statistical Society, 1995.

GGSEA analysis were performed using GSEA v3.0 tool developed by the Broad Institute.

Transcriptomic data have been deposited at the Gene Expression Omnibus database (GSE130625).

### Tumor xenografts in NOD/SCID IL-2 receptor g-chain-null mice

Xenograft tumors in NOD/SCID IL-2 receptor g-chain-null mice (NSG) mice of 6–8 years old were generated by injecting 2 × 10^6^ TF-1 KIT^D816V^ into the tail vein. Ten days after injection, mice were randomly treated either by adding doxycycline (200 μg/ml) and sucrose (10 μg/ml) to the drinking water or by adding sucrose only. Without blinding, human-cell engraftment (hCD45+ cells) into the bone marrow or spleen was evaluated by flow cytometry (BD FACS Calibur). Human cells were also isolated by magnetic sorting with hCD45 beads (Miltenyi Biotech, France) and analyzed by western blotting using the appropriate antibodies. Xenograft tumors in NOD/SCID mice were generated by subcutaneously injecting 2 × 10^6^ cells in 100 µL of PBS cells into both flanks. Once the tumors had reached 50−100 mm^3^ in size, without blinding the animals were given 2 mg/mL or 4 mg/mL of Stattic or vehicle (PBS). Tumor dimensions were measured with a caliper on the every 2 or 3 days and volume (*v*) was calculated using the formula: *v* = A*B^2^/2, where *A* is the larger diameter and *B* is the smaller diameter. All experiments were conducted in accordance with the guidelines of the Association for Assessment and Accreditation of Laboratory Animal Care International.

### Statistical analyses

Data from at least three independent experiments are reported as their means ± SEM. Statistical analyses were assessed using unpaired two-tailed Student’s *t*-test using Prism 5 software (GraphPad Software, Inc., La Jolla, CA, USA). A value of *p* < 0.05 was regarded as being significant: *, **, and *** correspond to *p* < 0.05, *p* < 0.01, and *p* < 0.001, respectively. The Kaplan−Meier method was used to estimate leukemia-free survival in xenografted mice. Log-rank *p*-values were used to compare leukemia-free survival among the subgroups.

## Supplementary information

Supplemental figures with legends
