# Supplementary material for: Oncogenic KIT mutations induce STAT3-dependent autophagy to support cell proliferation in acute myeloid leukemia
Source: Oncogenesis. 2019 Jul 16;8(8):39. doi: 10.1038/s41389-019-0148-9 (PMC6635375; doi:10.1038/s41389-019-0148-9)
Supplement: Supplementary file 1 — Supplemental figures with legends [file 41389_2019_148_MOESM1_ESM.pdf]

Figure S1

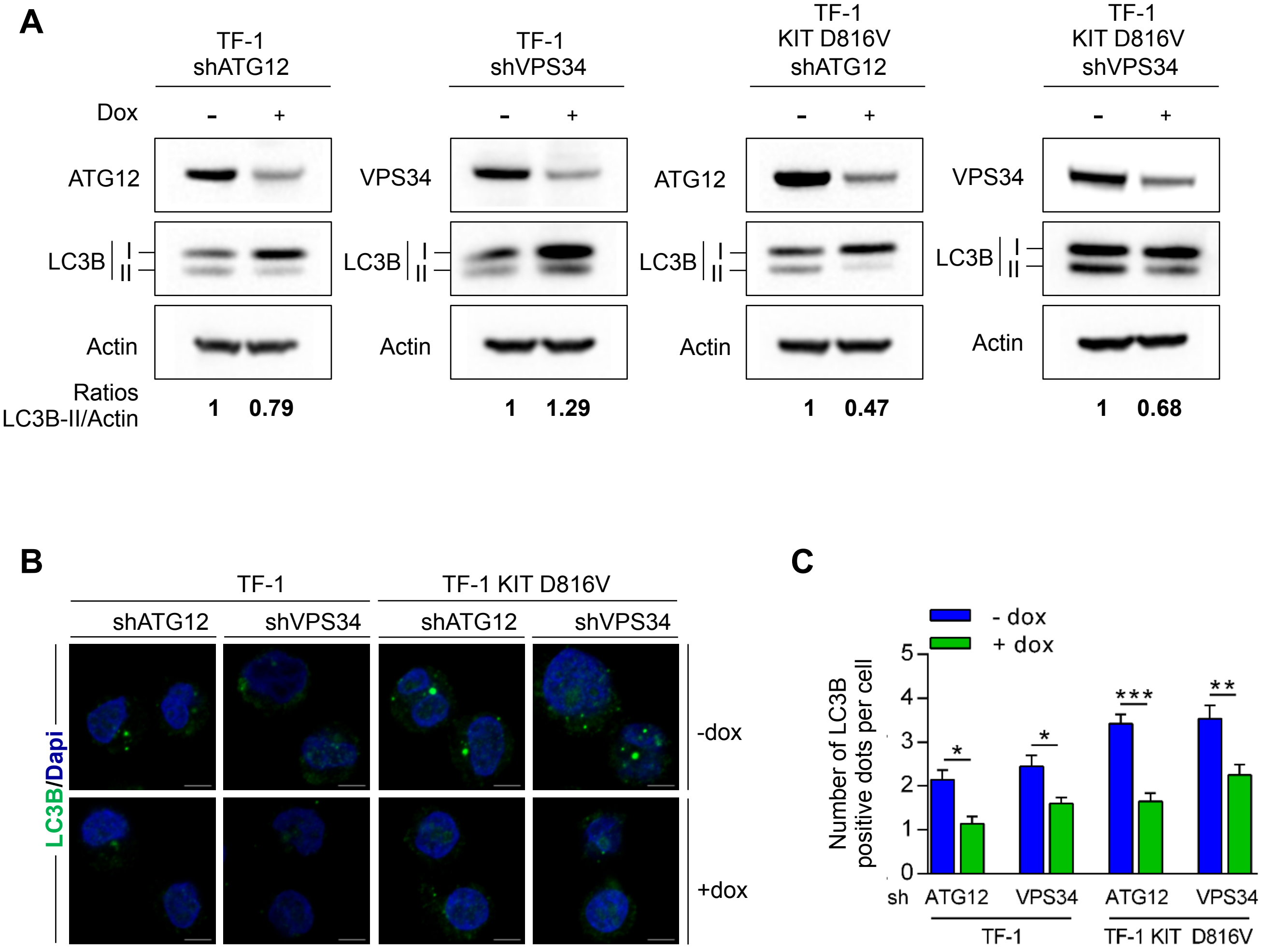
A
TF-1 shATG12
TF-1 shVPS34
TF-1 KIT D816V shATG12
TF-1 KIT D816V shVPS34
Dox - +
ATG12
VPS34
LC3B I II
Actin
Ratios LC3B-II/Actin
1 0.79
1 1.29
1 0.47
1 0.68
B
TF-1
TF-1 KIT D816V
shATG12
shVPS34
LC3B/Dapi
-dox
+dox
C
- dox
+ dox
Number of LC3B positive dots per cell
0
1
2
3
4
5
*
*
***
**
sh ATG12 VPS34 ATG12 VPS34
TF-1
TF-1 KIT D816V

Figure S2

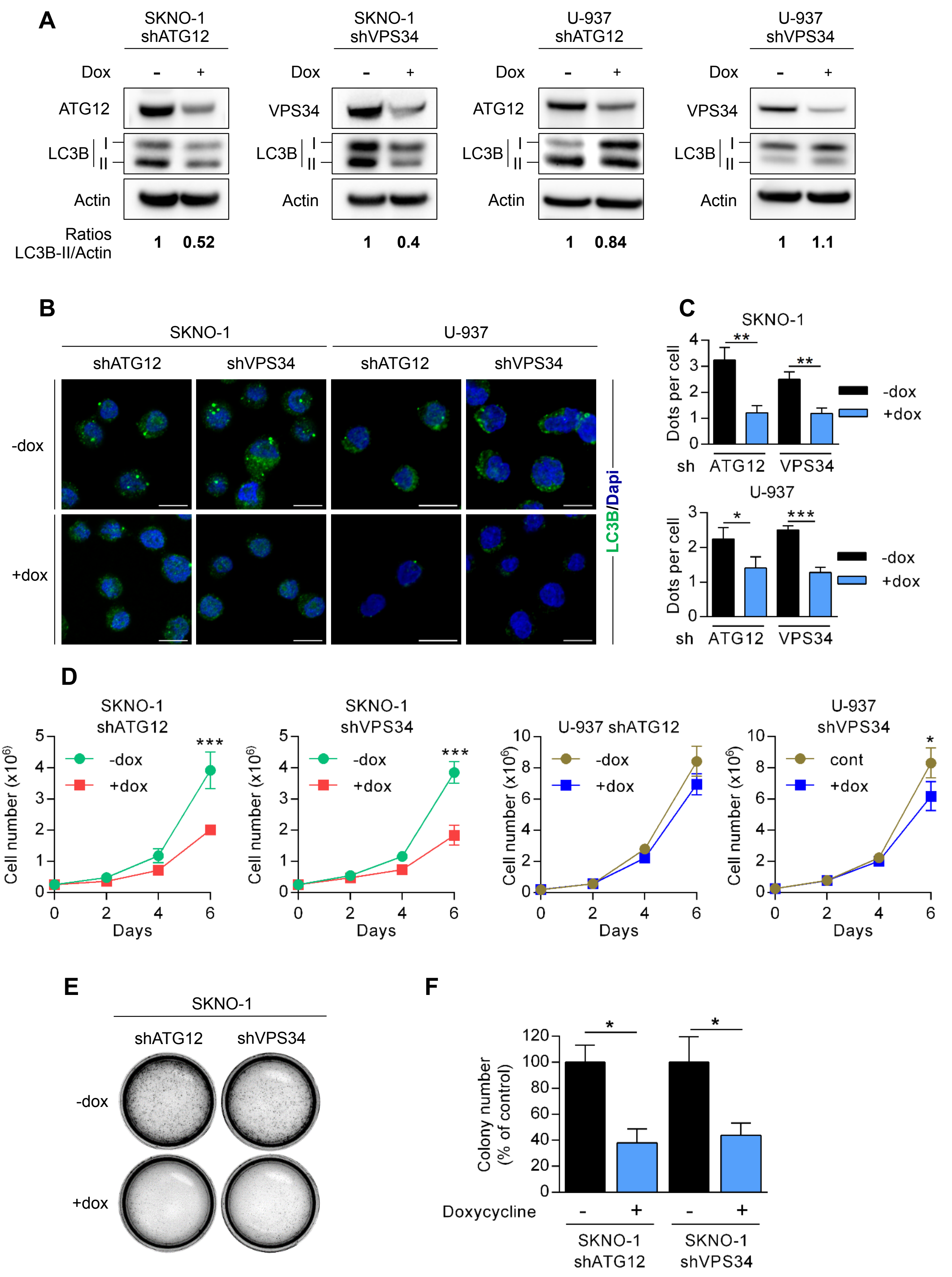
A
SKNO-1 shATG12
SKNO-1 shVPS34
U-937 shATG12
U-937 shVPS34
Dox - +
ATG12
VPS34
LC3B I II
Actin
Ratios LC3B-II/Actin
1 0.52
1 0.4
1 0.84
1 1.1
B
SKNO-1
U-937
shATG12
shVPS34
-dox
+dox
LC3B/Dapi
C
SKNO-1
U-937
Dots per cell
-dox
+dox
sh ATG12 VPS34
**
***
*
D
SKNO-1 shATG12
SKNO-1 shVPS34
U-937 shATG12
U-937 shVPS34
Cell number (x10^6)
Days
-dox
+dox
cont
E
SKNO-1
shATG12
shVPS34
-dox
+dox
F
Colony number (% of control)
Doxycycline - + - +
SKNO-1 shATG12
SKNO-1 shVPS34

Figure S3

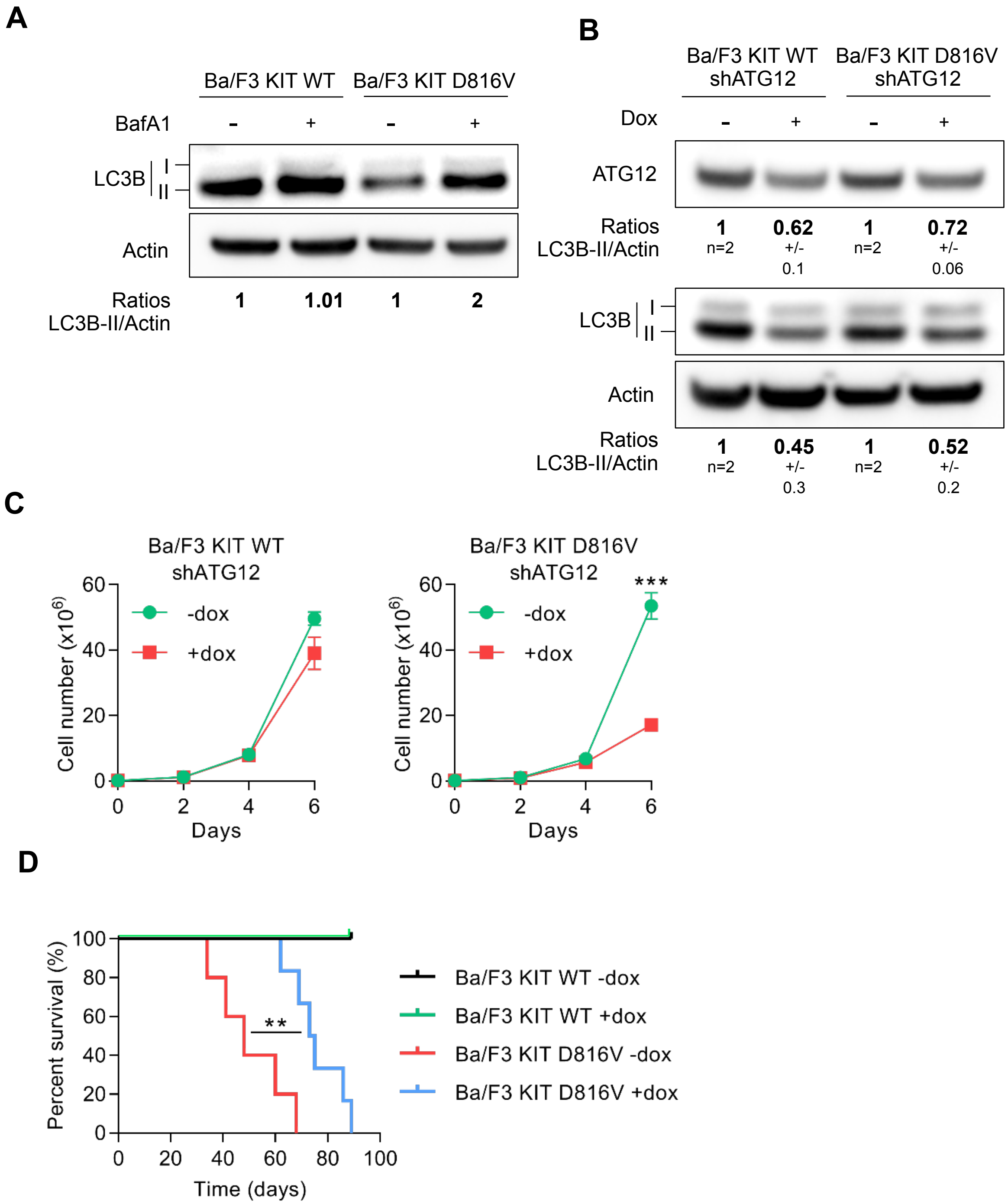
A
Ba/F3 KIT WT
Ba/F3 KIT D816V
BafA1
-
+
-
+
LC3B
I
II
Actin
Ratios
LC3B-II/Actin
1
1.01
1
2
B
Ba/F3 KIT WT shATG12
Ba/F3 KIT D816V shATG12
Dox
-
+
-
+
ATG12
Ratios
LC3B-II/Actin
1
n=2
0.62
+/-
0.1
1
n=2
0.72
+/-
0.06
LC3B
I
II
Actin
Ratios
LC3B-II/Actin
1
n=2
0.45
+/-
0.3
1
n=2
0.52
+/-
0.2
C
Ba/F3 KIT WT shATG12
Ba/F3 KIT D816V shATG12
Cell number (x10^6)
-dox
+dox
***
Days
D
Percent survival (%)
Time (days)
**
Ba/F3 KIT WT -dox
Ba/F3 KIT WT +dox
Ba/F3 KIT D816V -dox
Ba/F3 KIT D816V +dox

Figure S4

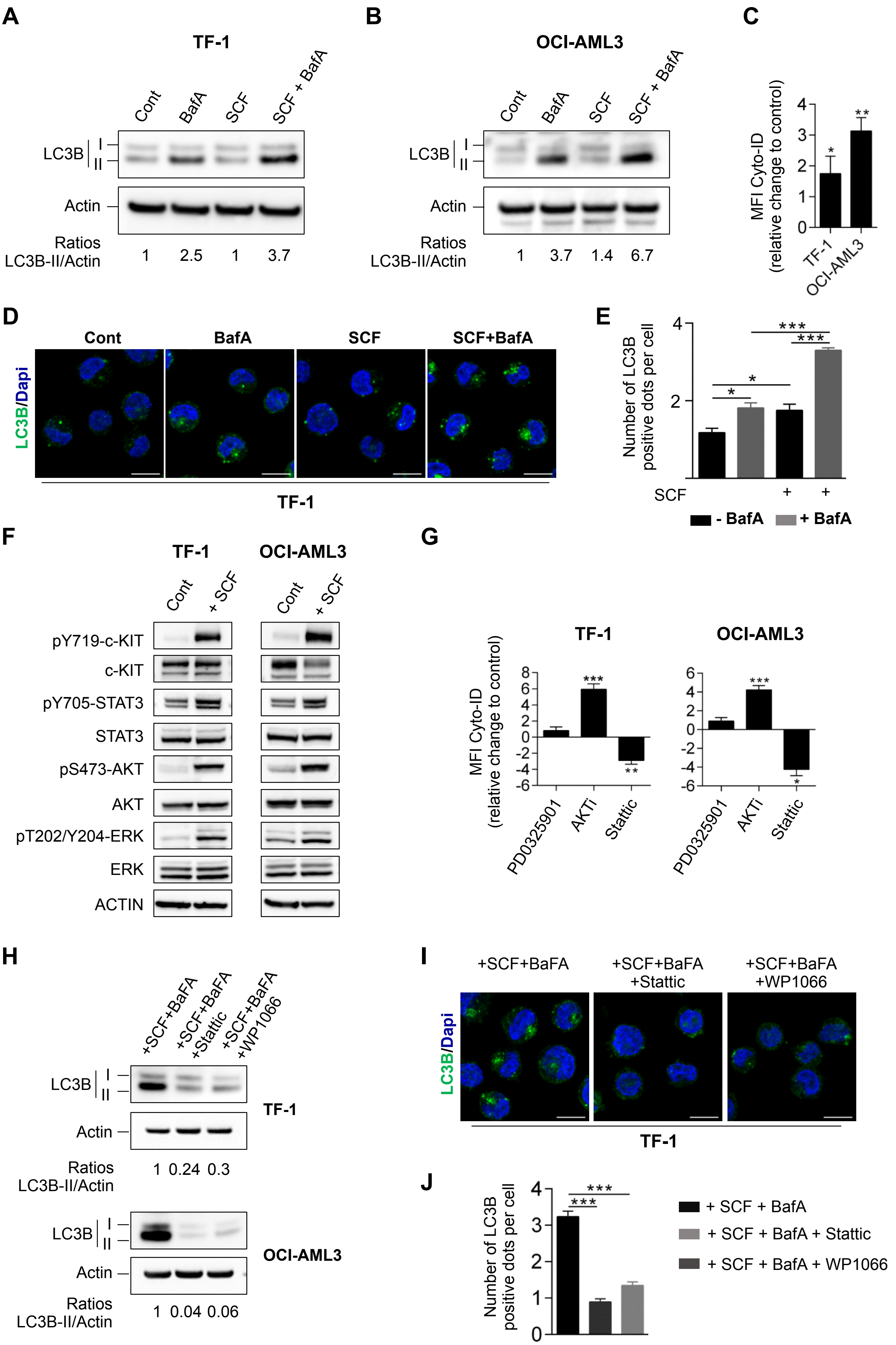
A
TF-1
Cont
BafA
SCF
SCF + BafA
LC3B
I
II
Actin
Ratios
LC3B-II/Actin
1
2.5
1
3.7
B
OCI-AML3
Cont
BafA
SCF
SCF + BafA
LC3B
I
II
Actin
Ratios
LC3B-II/Actin
1
3.7
1.4
6.7
C
MFI Cyto-ID
(relative change to control)
TF-1
OCI-AML3
D
Cont
BafA
SCF
SCF+BafA
LC3B/Dapi
TF-1
E
Number of LC3B
positive dots per cell
SCF
- BafA
+ BafA
F
TF-1
OCI-AML3
Cont
+ SCF
pY719-c-KIT
c-KIT
pY705-STAT3
STAT3
pS473-AKT
AKT
pT202/Y204-ERK
ERK
ACTIN
G
TF-1
OCI-AML3
MFI Cyto-ID
(relative change to control)
PD0325901
AKTi
Stattic
H
+SCF+BafA
+SCF+BafA +Stattic
+SCF+BafA +WP1066
LC3B
I
II
Actin
TF-1
Ratios
LC3B-II/Actin
1 0.24 0.3
LC3B
I
II
Actin
OCI-AML3
Ratios
LC3B-II/Actin
1 0.04 0.06
I
+SCF+BafA
+SCF+BafA +Stattic
+SCF+BafA +WP1066
LC3B/Dapi
TF-1
J
Number of LC3B
positive dots per cell
+ SCF + BafA
+ SCF + BafA + Stattic
+ SCF + BafA + WP1066

Figure S5

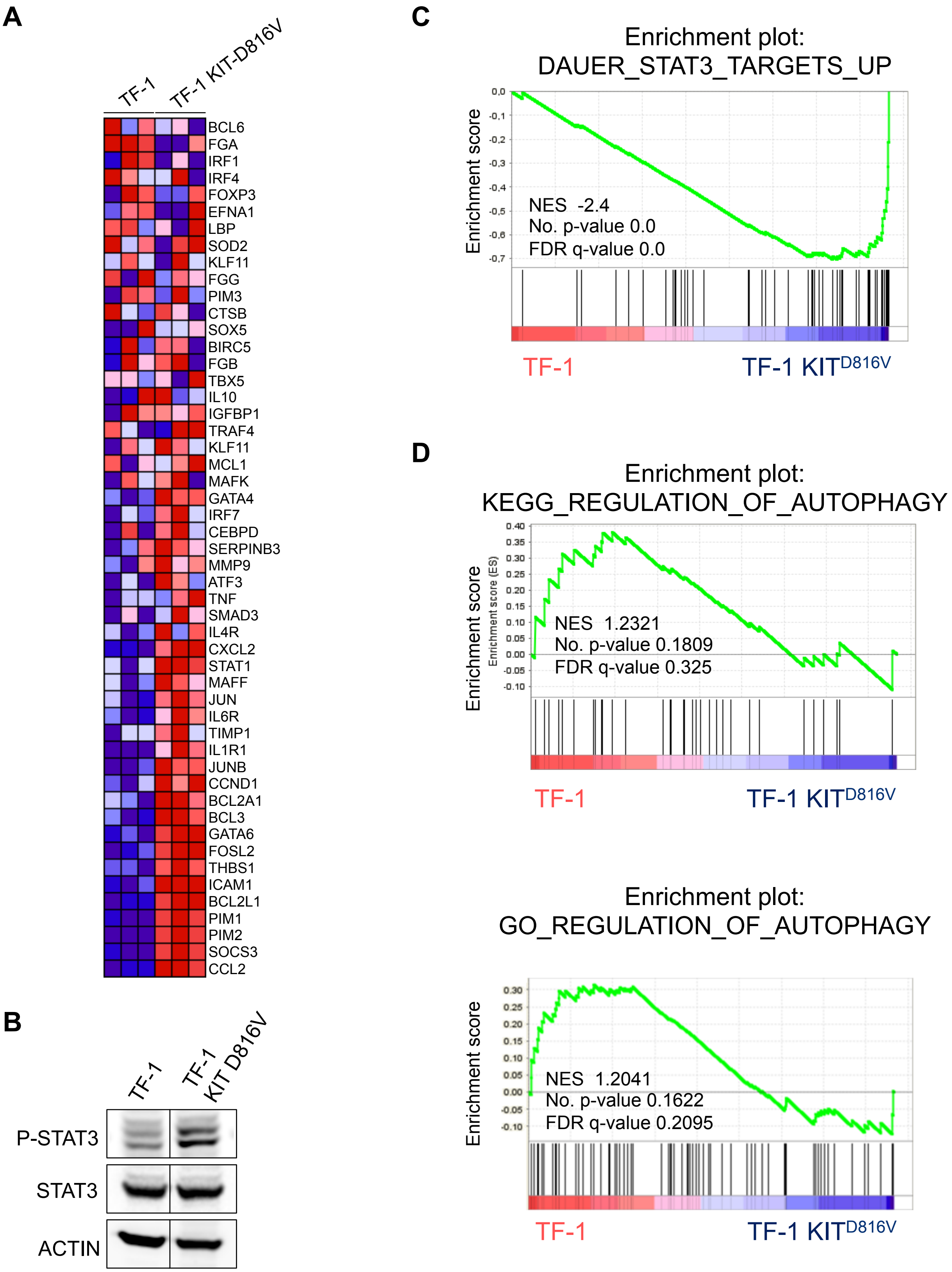
A
TF-1
TF-1 KIT-D816V
BCL6
FGA
IRF1
IRF4
FOXP3
EFNA1
LBP
SOD2
KLF11
FGG
PIM3
CTSB
SOX5
BIRC5
FGB
TBX5
IL10
IGFBP1
TRAF4
KLF11
MCL1
MAFK
GATA4
IRF7
CEBPD
SERPINB3
MMP9
ATF3
TNF
SMAD3
IL4R
CXCL2
STAT1
MAFF
JUN
IL6R
TIMP1
IL1R1
JUNB
CCND1
BCL2A1
BCL3
GATA6
FOSL2
THBS1
ICAM1
BCL2L1
PIM1
PIM2
SOCS3
CCL2
B
TF-1
TF-1 KIT D816V
P-STAT3
STAT3
ACTIN
C
Enrichment plot:
DAUER_STAT3_TARGETS_UP
Enrichment score
NES -2.4
No. p-value 0.0
FDR q-value 0.0
TF-1
TF-1 KIT^{D816V}
D
Enrichment plot:
KEGG_REGULATION_OF_AUTOPHAGY
Enrichment score
Enrichment score (ES)
NES 1.2321
No. p-value 0.1809
FDR q-value 0.325
TF-1
TF-1 KIT^{D816V}
Enrichment plot:
GO_REGULATION_OF_AUTOPHAGY
Enrichment score
NES 1.2041
No. p-value 0.1622
FDR q-value 0.2095
TF-1
TF-1 KIT^{D816V}

SUPPLEMENTARY FIGURE LEGENDS

Figure S1: Inhibition of autophagy by shRNA in TF-1 and TF-1 KIT^{D816V} cells. *A*. TF-1 or TF-1 KIT^{D816V} cells were incubated for 3 days with doxycycline to induce expression of shRNA against ATG12 or VPS34. Cells were then analyzed by western blotting. Numbers represent the LC3B-II/actin ratios obtained by densitometric analysis. *B.* LC3 staining, fluorescent labeling, and immunofluorescence analysis of cells expressing inducible shRNA. *C*. Quantification of LC3-positive autophagosomes (n=3 +/-sem).

Figure S2: Inhibition of autophagy in SKNO-1 and U-937 cells. *A-C.* Validation of shRNA against ATG12 and VPS34. *A*. SKNO-1 or U-937 cells were incubated for 3 days with doxycycline to induce expression of shRNA against ATG12 or VPS34. Cells were then analyzed by western blotting. Numbers represent the LC3B-II/actin ratios obtained by densitometric analysis. *B*. LC3 staining, fluorescent labeling, and immunofluorescence analysis of cells expressing inducible shRNA. *C*. Quantification of LC3-positive autophagosomes (n=3 +/-sem). *D*. Effects of shRNA on cell proliferation, measured by trypan-blue exclusion (n=3 +/-sem). *E-F*. Colony-forming cell (CFC) assays. *E*. shRNA-expressing cells were grown in methylcellulose medium in the presence of vehicle or doxycycline (5 µg/mL). *F*. Quantification of colonies (n=3 +/-sem).

Figure S3: Inhibition of autophagy in Ba/F3 hKIT^{D816V} cells. *A*. Oncogenic hKIT^{D816V} drives autophagy. *A*. Ba/F3 hKIT^{WT} or Ba/F3 hKIT^{D816V} cells were incubated for 2 h with PBS, or bafilomycin A1 (20 nM), and were then analyzed by immunoblotting using the appropriate antibodies. Numbers represent the LC3B-II/actin ratios obtained by densitometric analysis (n=1). *B*. Validation of shRNA against ATG12. *B.* Ba/F3 hKIT^{WT} or Ba/F3 hKIT^{D816V} cells were incubated for 3 days with doxycycline to induce expression of shRNA

against ATG12. Cells were then analyzed by western blotting. Numbers represent the ATG12/actin and LC3B-II/actin ratios obtained by densitometric analysis (n=2). *C*. Effects of shRNA on cell proliferation (n=3 +/-sem). *D*. Effects of shRNA on leukemic development. Ba/F3 hKIT^{D816V} cells expressing inducible shRNA against ATG12 were injected into the vein tails of NSG mice, doxycycline was added on day 10 and overall mouse survival was monitored (n=5-7 mice per group).

Figure S4: STAT3 drives autophagy in KIT expressing cells upon ligand activation. *A-E*. Activated KIT drives autophagy. *A-B*.TF-1 cells or OCI-AML3 were incubated or not with stem cell factor (100 ng/ml) for two hours +/- Bafilomycin A1 and were analyzed by immunoblotting using the appropriate antibodies. Numbers represent the LC3B-II/actin ratios obtained by densitometric analysis. *C*. Cells were incubated for 8 h with vehicle or 20 µM of chloroquine +/- SCF and then incubated with Cyto-ID before flow cytometry (n=3+/-sem). *D*. Cells were treated with vehicle or BafA1 +/- SCF for 2 h before LC3 staining, fluorescent labeling, and immunofluorescence analysis. *E*. Quantification of LC3-positive autophagosomes (n=3 +/-sem) from pictures obtained in D. *F*. Signaling pathways induced upon SCF stimulation in TF-1 and OCI-AML3 cells. Cells were incubated or not with SCF (100 ng/ml) for 10 min and were analyzed by immunoblotting using the appropriate antibodies. *G-J*. Identification of the signaling pathways involved in SCF stimulated KIT-induced autophagy. *G*. Autophagic flux was assessed by flow cytometry after Cyto-ID labeling. TF-1 and OCI-AML3 cells were incubated with 10 µM of chloroquine alone or in combination with PD03259 (100 nM, n=3+/-sem), Akt inhibitor-VIII (1 µM, n=3+/-sem), or Stattic (10 µM, n=3 +/-sem). *H*. Autophagic flux was evaluated by western blotting. TF-1 and OCI-AML3 cells were treated for 2 h with PBS and BafA1 at 20 nM alone or in association with the indicated inhibitors. STAT3 inhibitor WP1066 was used at 15 µM and Stattic at 10

μM. Numbers represent the LC3B-II/actin ratios obtained by densitometric analysis (n=1). *I.* Cells were treated for 2 h with vehicle, BafA1, Stattic, or WP1066 alone or in combination with BafA1 before LC3 staining, fluorescent labeling, and immunofluorescence analysis. *J.* Quantification of LC3-positive autophagosomes (n=3 +/-sem) from pictures obtained in I.

Figure S5: STAT3 controls autophagy through a transcription-independent mechanism. *A-B* KIT^{D816V} expression leads to STAT3 activation and to STAT3-regulated genes overexpression. *A.* Upregulated (red) and downregulated (blue) genes were generated from transcriptomes of TF-1 and TF-1 KIT^{D816V} AML cells (n=3). *B.* Comparison of STAT3 activation in TF-1 and TF-1 KIT^{D816V} cells by western blot with the indicated antibodies. *C-D.* Gene Set Enrichment Analysis (GSEA) performed using transcriptomes of TF-1 and TF-1 KIT^{D816V} cells. *C.* GSEA of STAT3 gene signature (DAUER_STAT3_TARGETS_UP). *D.* GSEA of two autophagy gene signatures (GO_REGULATION_OF_AUTOPHAGY; KEGG_REGULATION_OF_AUTOPHAGY).
